# Workgroup Report: Developing Environmental Health Indicators for European Children: World Health Organization Working Group

**DOI:** 10.1289/ehp.9958

**Published:** 2007-05-10

**Authors:** Kathy Pond, Rokho Kim, Maria-Jose Carroquino, Philippe Pirard, Fiona Gore, Alexandra Cucu, Leda Nemer, Morag MacKay, Greta Smedje, Antonis Georgellis, Dafina Dalbokova, Michal Krzyzanowski

**Affiliations:** 1 Robens Centre for Public and Environmental Health, University of Surrey, Guildford, United Kingdom; 2 WHO European Centre for Environment and Health, Bonn, WHO Regional Office for Europe; 3 Instituto de Salud Carlos III, Madrid, Spain; 4 Institut de Veille Sanitaire, Saint-Maurice, France; 5 WHO headquarters, Geneva, Switzerland; 6 Ministry of Health, Bucharest, Romania; 7 WHO European Centre for Environment and Health, Rome, WHO Regional Office for Europe; 8 European Child Safety Alliance, Amsterdam, The Netherlands; 9 Department of Occupational and Environmental Medicine, Uppsala University Hospital, Uppsala, Sweden; 10 Occupational and Environmental Health Department, Stockholm County Council, Stockholm, Sweden

**Keywords:** children, environmental health, Europe, indicators, policy

## Abstract

A working group coordinated by the World Health Organization developed a set of indicators to protect children’s health from environmental risks and to support current and future European policy needs. On the basis of identified policy needs, the group developed a core set of 29 indicators for implementation plus an extended set of eight additional indicators for future development, focusing on exposure, health effects, and action. As far as possible, the indicators were designed to use existing information and are flexible enough to be developed further to meet the needs of policy makers and changing health priorities. These indicators cover most of the priority topic areas specified in the Children’s Environment and Health Action Plan for Europe (CEHAPE) as adopted in the Fourth Ministerial Conference on Health and Environment in 2004, and will be used to monitor the implementation of CEHAPE. This effort can be viewed as an integral part of the Global Initiative on Children’s Environmental Health Indicators, launched at the World Summit on Sustainable Development in 2002.

Approximately a quarter of the global burden of disease can be attributed to environmental factors (Prüss-Ustün and Corvalán 2006). Children < 5 years of age bear > 40% of this burden [Smith et al. 1999; World Health Organization (WHO) 2002a]. Contaminated air, food, and drinking water are particular environmental factors affecting children in developing regions of the world (Abalak et al. 2001; Smith et al. 2000). An estimated 1.7 million deaths per year globally are attributed to unsafe water, sanitation, and hygiene; nine of 10 of these deaths occur in children, and nearly all of these occur in developing countries (Prüss-Ustün and Corvalán 2006). Although the traditional infectious disease threats to children’s health have largely been controlled in most industrialized countries by advances in water treatment, immunizations, waste disposal, and the provision of adequate food (Suk et al. 2003), diseases such as asthma and cancers including leukemia, learning disabilities, and congenital malformations are increasing in children in western Europe (Landrigan et al. 1998; Richardson et al. 2005; Simoni et al. 2005). Even if most of the deterministic processes leading to these diseases are multifactoral, there is increasing evidence that these diseases are influenced by environmental factors. Exposure to air pollution, lead, chemicals, and noise has been shown to impair children’s health and their cognitive development (Bellinger 2004; Niemann et al. 2005; Schwartz 2004). Despite the fact that the European Region contains some of the world’s wealthiest countries, widening health inequalities remain the principal determinant of mortality (Anonymous 2005), illustrated by the fact that almost 140 million (16%) people in the WHO European Region do not have a household connection to a drinking-water supply, 85 million (10%) do not have improved sanitation, and > 41 million (5%) do not have access to a safe drinking-water supply (Anonymous 2005). From a burden perspective, injury is responsible for 23% of all deaths and 19% of disability-adjusted life-years (DALYs) in 0- to 19-year-olds in the WHO European Region and has the largest environmental burden for children compared with outdoor/indoor contaminants, water sanitation and hygienic issues, or lead contaminants (Valent et al. 2004)

The Fourth Ministerial Conference on Environment and Health, held in Budapest, Hungary, in June 2004 (“The Budapest Conference”), focused on “the future for our children,” recognizing the need to address the rights of children, their health, and their particular vulnerability toward environmental risks, as well as to respond to emerging environmental concerns. The Declaration from the conference reaffirmed that the Environment and Health Information System (EHIS) is an essential tool for policy making relevant to children’s environmental health (bWHO Regional Office for Europe 2004b).

The Budapest Conference through its Declaration adopted the Children’s Environment and Health Action Plan for Europe (CEHAPE), an international instrument negotiated with member states to develop and manage environmental health indicators. CEHAPE sets four regional priority goals (RPGs) that encapsulate key themes for action on children’s health in relation to environmental factors: *a*) gastrointestinal health related to safe water and adequate sanitation; *b*) healthy and safe transport, mobility, and home environment to reduce injuries and enhance physical activity; *c*) respiratory health and clean air; and *d*) health through environment free of hazardous chemicals, physical, and biological factors.

Although the RPGs do not explicitly cover social indicators, the CEHAPE recognizes that these factors are critical in determining a child’s possible increased exposure or vulnerability to a number of environmental factors.

Reliable information is essential for prioritizing actions related to environmental exposures and their health effects as well as for monitoring the effectiveness of the actions taken. Currently, this information is widely scattered and difficult to obtain on international and national levels. Where it does exist, its contents and format are often inappropriate for international comparisons, for policy support, or for public communication. Providing decision makers with appropriate information regarding health effects attributable to environmental risks is of crucial importance. They require information about the issues of concern and an indication of the hazards and the risks that need to be addressed (Briggs 2003). Such information should enable them to assess the implications of their decisions, compare the potential effects of different decisions and choices, and ultimately develop effective prevention strategies (Corvalán et al. 2000). Such information includes environmental quality guidelines based on epidemiologic and toxicologic studies (e.g., WHO air quality guidelines; WHO 2006a). Overall, the information needs to be clear, concise, relevant, and powerful (Briggs 2003).

WHO has been coordinating the development of methods and tools for a pan-European EHIS to support policy making since 1999. In particular, the development of environmental health indicators—the EHIS central element—has been significantly advanced through a series of projects in collaboration with relevant international organizations. The project Development of Environment and Health Indicators for European Union (EU) countries (ECOEHIS), co-funded by the Directorate-General for Health and Consumer Protection of the European Commission (EC) and coordinated by WHO, was a part of this process and resulted in the proposal of 17 core indicators under six themes for monitoring the EU population’s exposure to environmental hazards, their health effects, and related policy actions (aWHO Regional Office for Europe 2004a; Kim et al. 2005).

The Declaration from the Budapest Conference reaffirmed that the EHIS is an essential tool for policy making relevant to children’s environmental health. The development and application of indicators focusing on children’s environmental health and facilitating monitoring and evaluation of the environmental health risks and the effect of interventions has become a significant objective (bWHO Regional Office for Europe 2004b).

An international project, Implementing Environment and Health Information System in Europe (ENHIS), co-funded by the EC and coordinated by the WHO Regional Office for Europe, developed a prototype of an evidence-based system to support children’s health and environmental policies in the European Region. Among the key products is a core set of children’s environmental health indicators to monitor the implementation of the CEHAPE with a prototype pan-European EHIS. Here we report the process and products of the ENHIS project related to developing children’s environmental health indicators.

## Methods

A working group comprised a core group of international experts representing each of the technical areas identified by the RPGs, plus a network of invited experts in each of the fields. This group carried out the following tasks: determine the needs of current and future environmental health policies; define the scope and target of the indicators; produce the methodologic guidelines for each of the indicators; pilot test the indicators and then further refine the indicators; and select a core set of indicators for pilot implementation. During the process, the group was concerned primarily with the need to select reliable indicators for which there was evidence in published literature that a clear health link exists between the environmental exposure and health outcome, while allowing comparison in the framework of the implementation of the CEHAPE. However, the group was mindful of the need not to place too much of a reporting burden on countries and therefore, where possible, to prioritize indicators for which routine monitoring and published data were readily available in most countries.

The indicators were designed to *a*) enable monitoring of children’s environmental health risks, their determinants, and effects of the intervention; *b*) provide appropriate information to countries to monitor the state of children’s environmental health, allow trends to be established, and support national policies and action programs; *c*) provide a sustainable basis for reporting and dissemination of evidence-based information (i.e., there is a policy need plus there is an established link between the exposure and health outcome) on children’s environmental health, avoiding duplication and ensuring continuity; and *d*) provide a basis for improvement of existing monitoring and surveillance systems by pointing out priority data gaps in order to inform policy-making decisions.

### Overall process of development of the indicators

Based on these criteria the process of development of the indicators was initiated. To present the links between environment, health outcomes, and actions the DPSEEA framework developed by Corvalán et al. (1996) was used. This framework defines driving forces (D), that lead to pressures on the environment (P), which in turn change the state of the environment (S), resulting in human exposures (Ex) and then to health effects (E). Actions (A) can be taken at any point during the chain to mitigate health effects.

**Figure 1:**
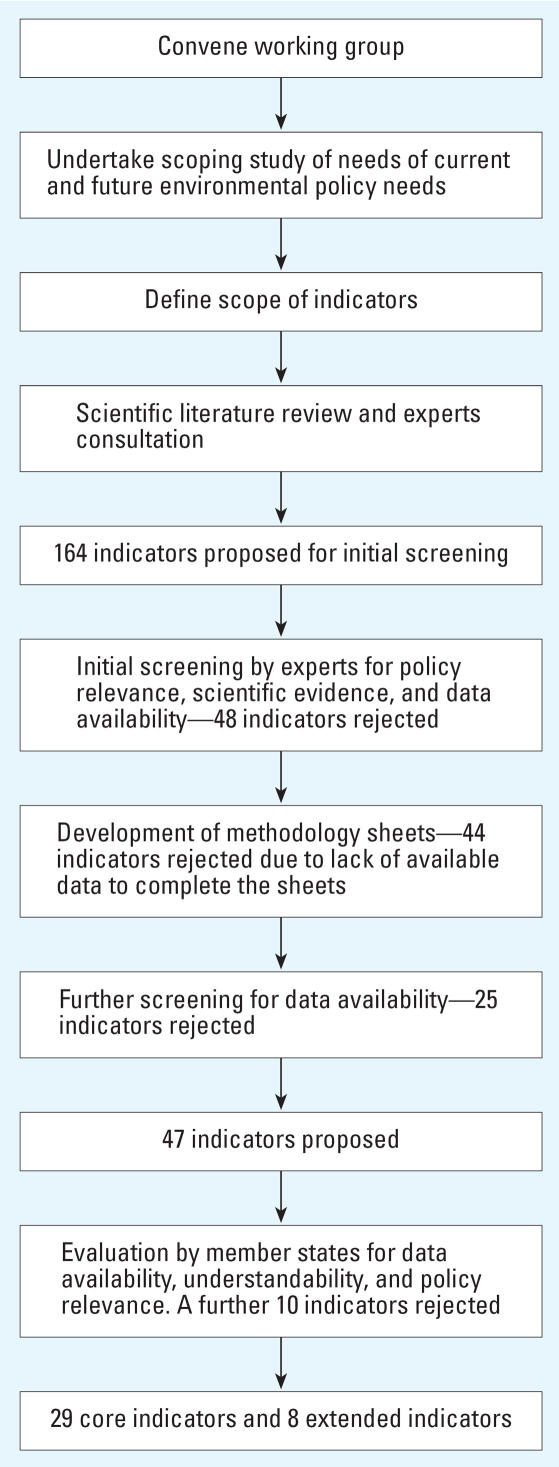
Overall process of development of the indicators.

The scope of indicators developed for the current project focused on exposure, health effects, and policy actions within the conceptual framework of cause-effect proposed by WHO (1999). The process of development is detailed in the following sections and summarized in Figure 1.

### Initial selection of candidate indicators

The working group undertook to assess the information needs of European environmental health policies by identifying the requirements of relevant legislation and guidelines such as the Protocol on Water and Health (cWHO Regional Office for Europe 2004c). This was done through the development of a questionnaire on current and planned children’s environmental and health policies at EU and domestic levels for the creation of an inventory. The questionnaire was sent to national collaborating centers of the ENHIS project and was completed by public health and environmental officials or national experts in the existing policies. The topics that were identified as policy priorities from this process were water and sanitation, noise, air pollution [including environmental tobacco smoke (ETS)], housing (including injuries), transport, and radiation. Social determinants were also considered important but these are not included in the key themes of CEHAPE, and it was eventually decided not to include social indicators in the project.

To address the assessment of the information needs of European environmental health policies, the working group reviewed the scientific literature of the links between environmental factors and health effects, and proposed a series of indicators of relevance to the RPGs regardless of data availability and existence of methodology sheets.

The review of the policy needs identified topic areas for which no clear regulatory framework exists. Examples include drinking-water safety, ensuring safe transport and mobility, counteracting obesity, and indoor air quality. The policy measures with clear legal and regulatory context are dedicated mainly to environmental protection and improvement of environmental quality. Furthermore, these policies do not cover the range of harmful health effects, particularly on children’s health, resulting from exposure to a regulated environmental substance.

These considerations guided the working group to select environmental public health thematic issues for which policy indicators needed to be developed. The working group sought to develop policy indicators to provide a snapshot of the measures put in place in countries to reduce and prevent hazardous exposures and related health effects in children. At the same time, the analysis of the policy indicators would identify policy gaps—areas not addressed by current policy measures.

Policy indicators were conceived as a composite index across a set of policy actions using a simple equal-weight linear model. To obtain the index, each individual policy measure was scored with the following options: 0 = not existing, 1 = partly existing, 2 = clearly stated and implemented across the country.

Because there is no consensus nor many systematic reviews on policy actions’ interventions, the working group checked international health regulation documents to select the policy components for the composite measure. These included the WHO Framework Convention on Tobacco Control (WHO 2003a), European Strategy for Tobacco Control (WHO Regional Office for Europe 2002), First Action Plan for Food and Nutrition Policy (WHO Regional Office for Europe 2001), European Child Safety Alliance (2004b), Child Safety Action Plan Project (European Child Safety Alliance 2004a), and the CEHAPE program and related table of actions (WHO Regional Office for Europe 2005).

This process resulted in 164 indicators (including those that had already been tested in the ECOEHIS project). The phase of reducing the number of indicators then began through a series of expert working group consultations. Initially, indicators that had already been tested and recommended by the ECOEHIS project and could be adjusted to meet the requirements of CEHAPE were selected. In addition, new indicators that corresponded to emerging policy and health priorities covered by the RPG action items of the CEHAPE were selected and developed. The proposed indicators were screened according to their policy relevance, health relevance, and potential data availability, including a review of published literature linking environmental factors and health outcomes as well as using the results from the policy questionnaire described above.

We assessed each indicator in terms of its credibility (i.e., based on a knowledge link between environment and health taking into account uncertainties), basic information on the definition, calculation method, interpretation, and potential data sources. The process and contents of assessments were recorded. There is scientific uncertainty in environmental health that needs to be reduced. During the process of selecting the indicators, we screened published literature to assess the scientific credibility of the available data. Within these criteria, the indicators were either set aside or accepted for development. This assessment reduced the number of proposed indicators to 116.

### Methodology sheets

To ensure the information collected on the proposed indicators was consistent and user friendly, we adopted a template for a methodology sheet used in the ECOEHIS project (Table 1).

Through the development of methodology sheets for each indicator, it became apparent that in the case of 44 indicators there were insufficient data available to continue development. These indicators were put aside, despite being considered potentially useful for the future.

To avoid duplication and assure continuity of developmental work, we reviewed the indicators tested and proposed in the ECOEHIS project for their relevance to children’s environmental health. Eleven indicators from the core indicators selected in the ECOEHIS project were adopted on the basis of their relevance to children’s health and the availability of data.

### Adjustment and screening of the indicators

Further review of the indicators was undertaken by member states and technical experts, until a final list of 29 core indicators was produced. The primary reason for rejecting proposed core indicators at this stage was unavailability of data from international sources. Nine indicators that were rejected from the core set were retained for future use and were termed “extended set.” These indicators were deemed highly relevant to children’s health, but at present the required data to compute the indicator do not exist.

Before finalization of the 29 core indicators, the experts were still uncertain about the feasibility and applicability of eight indicators that had not been evaluated in the ECOEHIS project. It was decided that these indicators should undergo an evaluation process in the countries represented in ENHIS (Austria, the Czech Republic, Finland, France, Germany, Hungary, the Netherlands, Poland, Romania, Spain). Four of these were action indicators and four were exposure indicators. The indicators screened were policies to promote safe mobility and transport for children; policies to reduce child unintentional injury unrelated to traffic accidents; policies to reduce child obesity; children living in homes using a hazardous source of fuel for cooking and heating; children living in proximity to heavily trafficked roads; children going to school with indoor air problems; actions to reduce children’s exposure to ultraviolet (UV) radiation; blood lead levels in young children. It was not deemed necessary to evaluate the indicators that had been developed for or adapted from the ECOEHIS project because these had already been tested. Details of the process taken to test the indicators selected for the ECOEHIS project are discussed by the WHO Regional Office for Europe (2004a).

The request to evaluate the indicators was sent to officials from the ministry of health and/or environment in the participating countries together with the methodology sheet and the contact data of the national partner institution. The questionnaire that accompanied the methodology sheets focused on four criteria of evaluating indicators and data elements: data quality, usefulness (combined as one category in Table 2 and described as understandability) data availability, and policy relevance (Table 2). The responses were collected using the questionnaire from April to June 2005.

## Results

### Screening in participating member states

Table 2 shows a summary of the results of the screening process in eight participating member states. The results revealed lack of data in four areas related to air pollution: the protection of children from air pollutants derived from cooking and heating facilities; the protection of children living in proximity to heavily trafficked areas; the protection of children going to schools with indoor air problems; and the protection of children from exposure to heavy metals such as lead (expressed as blood lead levels in young children). In addition, limited data were available in relation to the indicators on actions to reduce children’s exposure to UV. However, their relevance to policy in Europe was considered to be high.

### Core set of indicators

Tables 3–6 show the final set of children’s environmental health indicators according to the RPGs. The core indicators were deemed policy relevant and readily available from international data sources with sufficient quality and comparability. The eight indicators listed under “extended set” were retained for future development and use.

## Discussion

The indicators developed in this project met a specific task identified by the Budapest Declaration: to address the environmental factors that most affect the health of European children (bWHO Regional Office for Europe 2004b). Through the development of these indicators, the project has helped identify and prioritize the environmental health issues that are widespread in the European Region.

The screening process undertaken by eight countries highlighted the national variations in data availability, policy relevance, and priorities. It became clear through this process that even in this small number of member states there are gaps in policies relating to some areas of children’s environmental health as well as available data. One such area is indoor air quality. However, indoor air is an important issue with respect to children’s environmental health specifically targeted in CEHPAE, and keeping such indicators was considered valuable to encourage efforts to collect relevant data. Although not all of the issues are a priority in all countries, and countries should therefore choose the indicators that best suit their priorities and conditions, including resources, when establishing their own environmental health information system, there is clearly a need to fill these gaps through the development of national or international data collection systems.

The next phase of the project (begun in November 2005) was to implement the indicators in the European Region. This is making it possible to monitor the effect of actions taken to address the environmental health issues affecting children using standardized methodologies for data collection, processing, and dissemination, allowing inter- and intra-country comparisons and time trend analysis.

In the long term, the overall goal is to maintain an active and up-to-date European database of environmental health policies and data, which would facilitate the development of harmonized and science-based environmental health policies across Europe and increase their accountability in population health terms. Differences between national policies will and should remain, but they should be based on different conditions and needs, rather than on the lack of information to assess their effectiveness and accountability.

The environmental health indicators developed in this project can be readily applied in most EU countries in monitoring the implementation of CEHAPE. The indicators will need to be reviewed and updated regularly to maintain flexibility and responsiveness. By outlining the priority data flows in a pan-European EHIS, the core indicators will provide guidelines for the reporting on the progress of realization of four RPGs of the CEHAPE.

The development of environmental health indicators to monitor the trends in the state of European children contributes toward the objectives of the Global Initiative on Children’s Environmental Health Indicators launched at the World Summit on Sustainable Development in 2002, initiated by and building on efforts of the U.S. Environmental Protection Agency (WHO 2002b). The indicators developed and made available through the regional pilot surveys as well as information from ongoing international surveys and reporting mechanisms will be part of the comprehensive evidence base toward healthy public policies to better protect the health of our children and the generations to come.

## Figures and Tables

**Table 1:** Template of the methodology sheet used to define the indicators.

Indicator	Position in DPSEEA chain
Issue	Specification of the environmental health issue as stated in the CEHAPE regional priority goals to which the indicator relates
Justification for this indicator	Describe the importance of this indicator in terms of the priorities of children’s environmental health considering the magnitude, severity, amenability, and public concerns of the problem, with special attention to CEHAPE action item. State the evidence linking exposure, effect, and policy actions. Specify how this indicator can effectively monitor the achievement or actions of CEHAPE regional priority goals Quote the relevant part from CEHAPE as a key justification, followed by a summary of scientific evidence and policy effectiveness
Definition of indicator	Detailed technical definition of the indicator. If there are subindicators, provide their definitions.
Underlying definitions and concepts	Definition of all terms and concepts involved in describing and constructing the indicator
Specification of data needed	List data elements needed to construct the indicator
Data sources, availability, and quality	Outline potential sources of data, and comment on their quality and characteristics in terms of the indicator. Where appropriate, indicate ways of obtaining data that are not readily available
Computation	Specify how the indicator is computed: i.e., how the data are analyzed/processed to construct the indicator. Where relevant, express the computation process mathematically, and define the terms used
Units of measurement	Specify the units of measurement used in presenting the indicator
Scale of application	Specify the potential scales of application or level of aggregation. The scale specified refers to the area across which the indicator can be used; for geographic comparisons, the indicator might be developed at lower levels of aggregation. Definitions: local (within a city or community); regional (within a subnational region); national (for a country); international (across several countries or globally)
Interpretation	Describe how the indicator may be interpreted in relation to the issue(s) specified
Linkage with other indicators	Describe the relationship between this and other indicators relating to the issue(s) specified, listing all indicators and their position in the DPSEEA chain
Related data, indicator sets, websites	List similar or related indicators, proposed or developed as part of other indicator sets
Policy/regulatory context	List and briefly explain any international policy or regulations in the forms of declaration, action plan, framework, treaty, directives related the issue that this indicator is dealing with
Reporting obligations	Describe whether the reporting of the data elements for this indicator is obliged for the member states by the international legislations or constitutions

**Table 2:** Summary of screening results.

	Austria	Czech Republic	France	Hungary	Netherlands	Poland	Romania	Spain
Policies to promote safe mobility and transport for children								
Data availability	X	X	X	X	X	X	X	X
Understandability	X	X	X	X	X		X	X
Policy relevance	X	X	X	X	X		X	X
Policies to reduce children’s unintentional injury unrelated to traffic accidents								
Data availability	X	X	X	X	X	X	X	X
Understandability	X	X	X		X	X	X	X
Policy relevance	X	X	X	X	X	X	X	X
Policies to reduce child obesity								
Data availability	X	X	X	X	X	X	X	X
Understandability	X	X	X	X		X	X	X
Policy relevance	X	X	X	X	X	X	X	X
Children living at home using a hazardous source of fuel for cooking or heating								
Data availability				X				
Understandability		X	X	X	X		X	
Policy relevance	X	X	X	X			X	
Children living in proximity to heavily trafficked roads								
Data availability				X	X			
Understandability	X	X	X	X	X	X	X	
Policy relevance	X	X	X	X	X	X	X	
Children going to schools with indoor air problems								
Data availability								
Understandability			X	X			X	
Policy relevance	X		X	X			X	
Actions to reduce children’s exposure to UV								
Data availability	X	X	X	X	X	X		
Understandability	X	X		X	X	X	X	X
Policy relevance	X	X	X	X			X	X
Blood lead levels in young children								
Data availability			X			X		
Understandability	X	X	X	X	X	X		X
Policy relevance	X		X	X	X			X

**Table 3:** Core and extended indicators related to CEHAPE regional priority goal I.

Indicator title (and type)	Origin and international data source, if available	Definition of the indicator
Core indicators		
Wastewater treatment (exposure)	Adapted from ECOEHIS	Percentage of the child population served by sewage connected to a wastewater treatment facility that produces a regulated effluent discharge monitored by the competent authorities, or to an alternative safe local wastewater disposal system, e.g., septic tank
Recreational water quality (exposure)	Adapted from ECOEHIS	Proportion of identified bathing waters, falling under the EU bathing water directive definition (CEC 1976)
Drinking-water compliance (exposure)	Adapted from ECOEHIS	Proportion of the drinking-water samples analyzed from regulated public supplies that fail to comply with the *Escherichia coli* parameter of the EU drinking-water directive (CEC 1998)
Safe drinking water (exposure/policy)	Adapted from ECOEHIS	Proportion of the child population with continuous access to an adequate amount of safe drinking water in the home
Management of bathing waters (policy)	Adapted from ECOEHIS	Percentage of identified bathing waters which are covered by management systems as described by WHO (2003b)
Water safety plans (policy)	Adapted from ECOEHIS	Proportion of the child population served by a potable water supply covered by a ‘water safety plan’ as described by WHO (2006b)
Extended set of indicators		
Reliability of the water supply (exposure)	New	Percentage of the child population who have access to a reliable water supply
Outbreaks of waterborne diseases in children (health)	New	Number of outbreaks of fecal–oral water-related illness in the child population reported separately for drinking-water and recreational waters
Incidence of priority diseases in children (health)	New	The incidence of key water-related infections in the child population

**Table 4:** Core and extended indicators related to CEHAPE regional priority goal II.

Indicator title (and type)	Origin and data source, if available	Definition of the indicator
Core indicators		
Child mortality from traffic accidents (health)	Amended from ECOEHIS	Child mortality from traffic accidents by age group and by mode of accident
Policies for safe transportation for children (policy)	Child Safety Action Plan (European Child Safety Alliance 2004a)	Existence and actual enforcement of legislation and regulations establishing mandatory requirements for safe mobility and transport for children
Children’s mortality due to unintentional injuries not related to traffic accidents (health)	Amended from ECOEHIS	Data available from the WHO Mortality Database (WHO 2005). Cause-specific child mortality rates per 100,000 population for unintentional injuries not related to traffic accidents
Policies to reduce children’s mortality due to unintentional injuries not related to traffic accidents (policy)	Child Safety Action Plan (European Child Safety Alliance 2004a)	Existence and enforcement of legislation and regulations aimed at reducing child injury
Prevalence of overweight and obesity in adolescents (health)	New. Data found in HBSC (Currie et al. 2004)	Percentage of adolescents 15–19 years of age who are adequate weight, overweight, or obese, where adequate weight is defined as a BMI < 25 kg/m^2^, overweight is defined as a BMI 25–30 kg/m^2^, obesity is defined as a BMI of ≥30 kg/m^2^
Percentage of physically active children (exposure)	New. Data available in HBSC (Currie et al. 2004)	The percentage of children reporting to be physically active for 1 hr/day at least 3 times per week
Policies to reduce childhood obesity (policy)	New	Composite index of the willingness and commitment to implement a national strategy to prevent obesity in accordance with the WHO Global Strategy on Diet, Physical Activity and Health (WHO 2004) and the WHO Food and Nutrition Action Plan for the WHO European Region, 2000–2005 (WHO Regional Office for Europe 2001)
Extended set of indicators		
Mode of child transportation to school (exposure)	New	Percentage of children going to school by different modes

Abbreviations: BMI, body mass index; HBSC, Health Behaviour in School-aged Children study.

**Table 5:** Core and extended indicators related to CEHAPE regional priority goal III.

Indicator (and type)	Origin and data source, if available	Definition of the indicator
Core indicators		
Policies to reduce tobacco smoke exposure in children (policy)	Adapted from ECOEHIS indicator	This indicator is aimed at constructing a composite index of capability for implementing policies to reduce smoking and exposure to ETS in children and adolescents
Prevalence of allergies and asthma in children (health)	New	Prevalence (%) of children with asthma in age groups (years) 0–4, 5–9, 10–14, 15–19 of total population of children in the respective age group Prevalence (%) of allergy toward house dust mites, pollens, furry animals, and molds
Infant mortality due to respiratory diseases (health)	New	Annual mortality rate due to respiratory diseases in children > 1 month and < 1 year of age
Children’s exposure to air pollutants (exposure)	Adapted from ECOEHIS indicator	PM_10_: Child population-weighted annual mean PM_10_ concentration PM_2.5_: Child population-weighted annual mean PM_2.5_ concentration O_3_: Child population-weighted annual mean (of maximum daily 8 hr means) O_3_ concentration NO_2_: Child population distribution of exceedance hours of air quality limit values SO_2_: Child population distribution of exceedance days of air quality values
Children living in homes with dampness problems (exposure)	Adapted from ECOEHIS indicator	Percentage of children 0–4, 5–9, 10–14, 15–19 years old living in damp housing This indicator uses the Eurostat SILC (variable HH040) on dampness-related problems such as *a*) leaking roof, *b*) damp walls/floors/foundations, and *c*) rot in window frames or floor; all of which could lead to or represent mold growth
Children exposed to tobacco smoke (exposure)	New	Percentage of children 0–4, 5–9, 10–14 years old daily exposed to ETS Percentage of smokers among children 10–14, 15–19 years old
Children living in homes using solid fuels (exposure)	New. Data from international surveys, e.g., demographic and health surveys (Measure DHS 2007), world health statistics (WHO 2006c), and censuses. Data also available from the Millennium Indicator Database (UN 2006) and Eurostat (2007)	Percentage of children 0–4, 5–9, 10–14 years old living in households using: coal, wood, dung, gas, or kerosene as the main source of heating and cooking fuel
Children living in proximity to heavily trafficked roads (exposure)	New	Percentage of children 0–4, 5–9, or 10–14 years old living in proximity to heavily trafficked roads
Extended set of indicators		
Hospital admissions and emergency room visits due to asthma in children (health)	New	No. of hospital admissions or emergency room visits for asthma per 1,000 children by age group
Children going to schools with indoor air problems (exposure)	New	Percentage of children going to schools or day care centers with moisture damage or mold growth during the year Percentage of children going to schools and day care centres with a ventilation < 7 L/sec per person

Abbreviations: ETS, environmental tobacco smoke; PM_2.5_, PM_10_, particulate matter < 2.5 or 10 μm in aerodynamic diameter.

**Table 6:** Core and extended indicators related to CEHAPE regional priority goal IV.

Indicator title	Origin and data source, if available	Definition of the indicator
Core indicators		
Children exposed to harmful noise at school (exposure)	New. Noise map available in 2008 according to EU directive on environmental noise (CEC 2002)	Percentage of children going to primary or secondary schools located in places that are considered to be exposed to transport (road, rail, and aircraft) noises > 55 dB (A) average during school hours
Actions to reduce children’s exposure to UV (policy)	New	This is a composite index of national efforts to improve protection of children against UV exposure
Incidence of melanoma (health)	Adapted from ECOEHIS. Data available from International Agency for Research on Cancer	Incidence of melanoma by age periods of 5 years, among children and adults up to 45–50 years of age
Incidence of childhood leukemia (health)	New	Annual incidence rate of leukemia
Work injuries among employees < 18 years of age (health)	New. Data available from EUROSTAT (Eurostat 2007)	Incidence rate of work accidents with victims < 18 years of age per 100,000 workers According to the severity, there are two subindicators: Nonfatal work injuries with > 3 days’ absence from work Fatal work injuries
Children’s exposure to chemical hazards in food (exposure/policy)	New. Data available from WHO (2007)	Dietary exposure assessment to potentially hazardous chemicals monitored in children’s food Global Environmental Monitoring System/Food Contamination Monitoring and Assessment Programme (GEMS/Food)
Persistent organic pollutants in human milk (exposure)	New. Data available from WHO (2007)	Concentrations of dioxins and polychlorinated biphenyls in human milk fat (expressed as WHO toxicity equivalents in pg/g) in pooled samples using standardized collection and analytical protocols established by WHO
Blood lead levels in children (exposure)	New	Average of blood lead levels (μg/dL) in children < 6 years of age Percentage of children < 6 years of age with elevated blood lead levels (> 10 μg/dL)
Extended set of indicators		
Radon levels in schools (exposure)		Distribution of annual radon levels in classrooms and inhabited rooms of kindergarten, schools, and colleges Estimated arithmetic mean, median of radon concentration Estimated percentage (and number) of classrooms and other rooms with annual mean levels of radon > 200, 400 Bq/m^3^ Specified at the national or regional level
Children with hearing loss and reporting tinnitus (health)		Proportion of children with hearing loss due to noise
